# Genetic polymorphisms and decreased protein expression of ABCG2 urate transporters are associated with susceptibility to gout, disease severity and renal-overload hyperuricemia

**DOI:** 10.1007/s10238-022-00848-7

**Published:** 2022-08-08

**Authors:** Márton Pálinkás, Edit Szabó, Anna Kulin, Orsolya Mózner, Rita Rásonyi, Péter Juhász, Krisztina Nagy, György Várady, Dóra Vörös, Boglárka Zámbó, Balázs Sarkadi, Gyula Poór

**Affiliations:** 1 National Institute of Locomotor Diseases and Disabilities, Frankel Leo str. 38-40, 1023 Budapest, Hungary; 2 Doctoral School of Molecular Medicine, Semmelweis University, Budapest, Hungary; 3 Institute of Enzymology, Research Centre for Natural Sciences, Budapest, Hungary; 4 Section of Rheumatology and Physiotherapy, Department of Internal Medicine and Haematology, Semmelweis University, Budapest, Hungary

**Keywords:** ABCG2 transporters, Genetics, Protein expression, Gout severity, Renal-overload hyperuricemia

## Abstract

Gout is a common crystal induced disease of high personal and social burden, characterised by severe arthritis and comorbidity if untreated. Impaired function of ABCG2 transporter is causative in gout and may be responsible for renal-overload type hyperuricemia. Despite its importance, there is limited information on how clinical parameters correlate with protein expression and that with genetic changes. Urate and clinical parameters of 78 gouty patients and healthy controls were measured among standardised circumstances from a Hungarian population. ABCG2 membrane expression of red blood cells was determined by flow cytometry-based method and SNPs of this protein were analysed by TaqMan-based qPCR. The prevalence of ABCG2 functional polymorphisms in gouty and control patients were 32.1 and 13.7%, respectively. Most common SNP was Q141K while one sample with R236X, R383C and the lately described M71V were found in the gouty population. These polymorphisms showed strong linkage with decreased protein expression while the latter was also associated with higher fractional urate excretion (FUE) and urinary urate excretion (UUE). This study firstly evaluated ABCG2 protein expression in a clinically defined gouty population while also proving its associations between ABCG2 genetic changes and renal-overload hyperuricemia. The paper also highlighted relations between ABCG2 SNPs, gout susceptibility and disease severity characterised by an early onset disease with frequent flares and tophi formation.

## Introduction

Gout is a common crystal-induced arthritis, characterised by chronic hyperuricemia leading to crystallisation and deposition of monosodium-urate crystals. As a result of crystal formation, erosive arthritis [1] and systemic comorbidities [2] occur, culminating in disability, negative socioeconomic changes and elevated mortality rates [3, 4]. The prevalence of gout shows regional differences affecting about 0.5–2.5% of the adult European population [5].

Besides common risk factors [6], genetics play an important role in the etiopathogenesis of gout [7]. According to genome-wide association studies (GWAS) and meta-analyses, genetic variations of the ABCG2 transporter are associated with the disease [8, 9]. ABCG2 is a member of the ATP-binding cassette (ABC) transporter family (subgroup G2). It is expressed in several locations, such as erythrocytes, blood–brain-barrier, placenta, liver, kidney and gut. The homodimeric transporters implement an ATP-dependent uric acid excretor activity through luminal transport mainly in the terminal ileum and proximal tubules of the kidney cortex [10, 11]. Besides, they have a key role in the elimination and modulation of xeno and endobiotics [12].

Approximately 30% of urate excretion is conducted through the intestines [13]. The evaluation of a (human) extrarenal urate transporter protein is difficult, as it can mostly be done via invasive sampling. However, with better understanding of extrarenal transport mechanisms in the past years, the classification of hyperuricemia and gout has been challenged. The classical overproduction–underexcretion approach is about to change into renal-overload (ROL) hyperuricemia (overproduction and extrarenal underexcretion types), and renal underexcretion type hyperuricemia. In this new classification, extrarenal underexcretion may refer mostly to decreased intestinal ABCG2 urate excretion [14].

ABCG2 is a well-studied urate transporter, while there is still limited information on how genetic changes correlate with protein expression in gouty patients. Previously, several studies have shown the association of gout with ABCG2 polymorphisms [15–17], the stronger effects of common dysfunctional ABCG2 variants in the risk of hyperuricemia/gout than major environmental risk factors such as age, obesity, and heavy drinking [18], as well as in the earlier onset of gout and the presence of a familial gout history [19, 20]. Moreover, ABCG2 dysfunction was reported as a strong independent risk for paediatric-onset hyperuricemia/gout [21] and the role of the ABCG2-Q141K mutation has been shown in younger hyperuricemic/gout individuals [22]. However, in this study we firstly evaluated in clinically defined gouty patients the wide spectrum of both the polymorphisms, the related ABCG2 protein expression levels and the clinical parameters.

Here we analysed the relation between the four most common clinically relevant functional single nucleotide polymorphisms (SNP) of ABCG2 gene in the studied population and the main clinical hyperuricemia parameters along with susceptibility and the characteristics of severe gout such as early onset of the disease, the number of occurring flares (flare/past 12 months) and the presence or absence of subcutaneous tophi [23, 24]. To overcome sampling difficulties, we used a recently developed flow cytometry method [25] to measure ABCG2 protein levels in gouty patients' red blood cell (RBC) membranes. We assessed how SNPs correlate to altered protein levels and whether these changes lead to a ROL-type hyperuricemia.

## Methods

### Study design and patients

In the prospectively recruited, non-interventional study we collected data on Hungarian gouty arthritis patients taken care of at the National Institute of Locomotor Diseases and Disabilities, Budapest, Hungary. Informed consent was obtained from all participants and the work was conducted in accordance with the Declaration of Helsinki and with the permission of the national Scientific and Research Ethic Committee of the Medical Research Council, Hungary (study number 41006-1/2013/EKU).

Clinical classification of gout was based on the EULAR criteria [26]. Overproducer type patients and those taking colchicine 3 months prior to enrolment were not included.

Healthy volunteers with no history of hyperuricemia or gout were enrolled as a control group.

### Urate and clinical parameters of gouty subjects

After signing informed consent forms, blood tests were taken from participants within the frame of their general examination, therefore no extra intervention was needed. Serum and urine uric acid were determined using Siemens Expansion Plus clinical chemistry analyser. The method is a modified form of the uricase method originally described by Bulger and Johns [27] and as well as by Kalckar [28]. The level of hyperuricemia was set at 7 mg/dl or over, whereas hyperuricosuria was defined as urinary urate excretion of 600 mg/die or over. In order to analyse hyperuricemia, serum urate level, fractional urate excretion (FUE) [urate clearance/creatinine clearance × 100] and urinary urate excretion (UUE) [mg urinary urate/24 h] were measured on the same day. Hyperuricemic patients were considered renal underexcretion type if FUE was 5.5% or below and overproducer type if FUE was > 10% and UUE was > 600 mg/die. We performed a 24 h urine collection after five days of low-purine diet, adequate hydration, and suspension of xanthine-oxidase inhibitors and diuretics. Urine collection was launched from the second urination and consisted of the first sample the morning 24 h later. All samples were saved and kept cold in a single container.

### Genetic analysis from genomic DNA

Genomic DNA was purified from 300 µL of EDTA-anticoagulated blood samples with Puregene Blood Kit (Qiagen). TaqMan-based qPCR reactions for SNP detection were performed in a StepOnePlus device (Applied Biosystems) with premade assay mixes (Q141K—cat. 4362691, M71V and R236X—cat. 4351379), or with custom-designed assay mixes (R383C), and master mix (cat. 4371353) from Thermo Fisher. TaqMan probe specificities were verified by sequencing [29].

### ABCG2 protein expression analysis

The measuring of ABCG2 levels in RBCs were carried out according to a recently developed flow cytometry-based method [25, 29, 30]. Blood samples were freshly collected (2–6 h before the flow cytometry analysis). We fixed and permeabilized the RBC membranes by using 1% formaldehyde solution, resulting in RBC “ghosts”. WGA-Alexa Fluor 647 (Thermo Fisher, cat. W32466, final cc. 1 µg/ml) were added to the fixed and washed RBC ghosts to separate them from debris during flow cytometry. Ghosts were incubated with the ABCG2-specific primary antibody (Bxp-34, mouse monoclonal antibody, Abcam, cat. ab3379) followed by a secondary, Alexa Fluor 488-labeled goat anti-mouse (H + L) antibody (Thermo Fisher, A-11001), in 96 well plates. Cellular fluorescence was measured twice each case by a FACSCanto II flow cytometer equipped with a plate loader. The agreement between the two measurements were tested by intraclass correlation coefficient (ICC). ICC estimates and their 95% confident intervals were calculated based on an absolute-agreement, 2-way model (package irr in R). Intraclass correlation coefficient (ICC) showed strong significant agreement between the two measurements of Bxp34 (ICC = 0.988, 95% confident intervals were: 0.982–0.991, *p* < 0.001). Therefore, we used the average of the two results for further analysis.

### Statistical methods

Statistical analysis was conducted in R 4.0.3. [31]. The significance level was set at *p* < 0.05. When comparing two categorical variables, the Fisher tests were used, e.g. when differences in ratio of males/females or the presence/absence of at least one ABCG2 SNP were compared between gouty and control patients, or when the presence of tophi and more than one flares were compared among gouty patients having or not having polymorphisms. The results of the Fisher tests gave us the odds ratios.

Separate general linear models were used to test the differences between gouty and control patients, or other categorical variables (such as gender, or presence/absence of at least one ABCG2 SNP) in regard to the following variables: age at the time of study, age at diagnosis, serum urate level, urinary urate excretion (UUE), fractional urate excretion (FUE) and red blood cell (RBC)-ABCG2 protein expression. For multiple comparison the Dunnett test was used.

Pearson correlation was used to test correlation among gouty patients UUE and FUE along with RBC-ABCG2 expression; the lines on the correlation plots were fitted by using general linear models.

## Results

We collected and analysed data of 151 patients, 78 gouty cases and 73 controls. The ratio of males was 91.0% among gouty and 86.3% among control patients, the difference was not significant. Mean (SD) age (age at the time of study) for control and gouty patients were 55.0 (13.3) and 57.4 (11.3) years, respectively. Age did not differ significantly among control and gouty patients or among males and females. The average (SD) serum urate level was significantly higher in gouty patients compared to control, 8.4 (1.6) vs 5.3 (1.0) mg/dl, (*p* < 0.001). Age and gender did not have a significant effect on serum urate level.

### ABCG2 genetic polymorphisms and susceptibility to gout

The most common SNP was Q141K (rs2231142) affecting 28.2% (*n* = 23) of gouty and 13.7% (*n* = 10) of control populations, appearing in hetero- and also homozygous form. There was one R236X (rs140207606) and one R383C (rs560659849) variant, both of them were gouty patients. One patient had two SNPs, being diagnosed with the recently identified [29] loss of function variant ABCG2-M71V (211A > G, rs148475733) and with a Q141K mutation. Altogether 23.2% (35/151) of the total population had at least one ABCG2 SNP (Table 1).

**Table 1:** Prevalence and most common types of ABCG2 polymorphisms in gouty and control patients

	Gouty patients, *n* = 78				Control, *n* = 73				European population [32]
	MAF	Homozyg	Heterozyg	WT	MAF	Homozyg	Heterozyg	WT	MAF
Q141K	0.16	2	21	55	0.08	1	9	63	0.09
M71V	0.01	0	1	77	0	0	0	73	0
R236X	0.01	0	1	77	0	0	0	73	0
R383C	0.01	0	1	77	0	0	0	73	0

One patient had Q141K/M71V form

*MAF* minor allele frequency; *Homozyg.* homozygous; *Heterozyg.* heterozygous; *WT* wild type

The prevalence of all studied ABCG2 SNPs in gouty and control patients were 32.1% (25/78) and 13.7% (10/73), respectively. The odds of having at least one ABCG2 polymorphism was three- times higher amongst gouty patients compared to control (odds ratio 3.0, 95% CI = 1.2–7.5, *p* = 0.012).

### Factors affecting disease severity in the gouty population

The average (SD) age at diagnosis of the disease, the prevalence of flare, tophi and the average (SD) UUE, FUE, serum urate level and RBC-ABCG2 protein expression in regard to the presence of ABCG2 SNPs in gouty patients can be seen in Table 2.

**Table 2:** Clinical and laboratory parameters of gouty patients with or without the studied ABCG2 polymorphisms

	Presence of studied ABCG2 polymorphisms in gouty patients, *n* = 25	Absence of studied ABCG2 polymorphisms in gouty patients, *n* = 53	*p* value
Mean ± SD age at diagnosis (years)	37.6 ± 11.8	45.7 ± 12.3	0.008
Presence of two or more flares during the past 12 months	20/25 (80.0%)	28/53 (52.8%)	0.026
Presence of tophi	10/25 (40.0%)	16/53 (30.2%)	0.445
Mean ± SD UUE (mg/24 h)	663.0 ± 190.1	522.7 ± 171.2	0.002
Mean ± SD FUE (%)	6.1 ± 1.8	4.6 ± 1.4	< 0.001
Mean ± SD serum urate level (mg/dl)	8.9 ± 1.9	8.3 ± 1.6	0.140
Mean ± SD relative RBC- ABCG2 protein expression (AU)	6.1 ± 1.5	7.8 ± 1.7	< 0.001

*UUE* urinary urate excretion; *FUE* fractional urate excretion; *RBC* red blood cell; *ABCG2* ATP-binding cassette (ABC) transporter family (subgroup G2); *AU* arbitrary units

Hyperuricemic patients were considered renal underexcretion type if FUE was 5.5% or below and overproducer type if FUE was > 10% and UUE was > 600 mg/die.

Gouty patients with functional ABCG2 SNPs have developed gout significantly earlier, with an estimated average (SE) of 8.1 (3.0) years in their life compared to gouty patients who did not have ABCG2 mutation/variants (*p* = 0.008).

Among those gouty patients who had at least one ABCG2 SNP, the odds of having two or more flares during the last 12 months was significantly, 3.5 times higher compared to wild type gouty patients (odds ratio 3.5, 95% CI = 1.1–13.8, *p* = 0.026).

However numerically there was a difference, the presence of subcutaneous tophi (40 vs 30.2%) and the serum urate level (8.9 vs. 8.3 mg/dl) was not influenced significantly by the presence of ABCG2 SNPs.

### Decreased ABCG2 protein expression in erythrocyte membrane

**Fig. 1:**
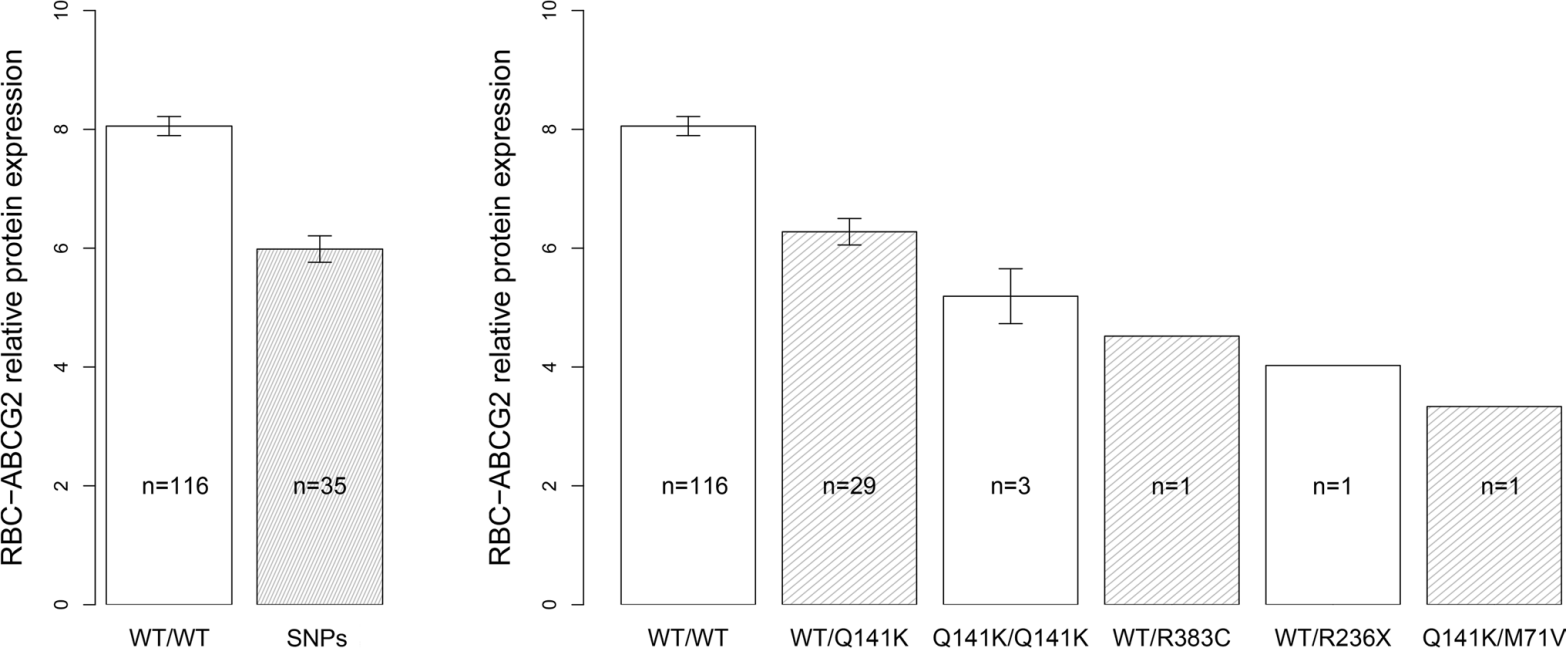
Correlation of functional ABCG2 polymorphisms and protein expression levels

RBC-ABCG2 protein expression levels were significantly lower, if the studied ABCG2 polymorphisms, either in hetero- or homozygous form, were present (Fig. 1a, b). The estimated decrease (SE) compared to wild type was 1.8 (0.3) for WT/Q141K (*p* < 0.001), 2.9 (0.9) for Q141K/Q141K (*p* = 0.014), 3.5 (1.6) for WT/R383C (*p* = 0.144), 4.0 (1.6) for WT/R236X (*p* = 0.068) and 4.7 (1.6) for Q141K/M71V (*p* = 0.021).

Mean values ± SD of erythrocyte ABCG2 relative protein expression levels in all wild type and all individuals with any SNP (A) or all wild type and individuals with specific SNPs (B).

### Renal-overload hyperuricemia

**Fig. 2:**
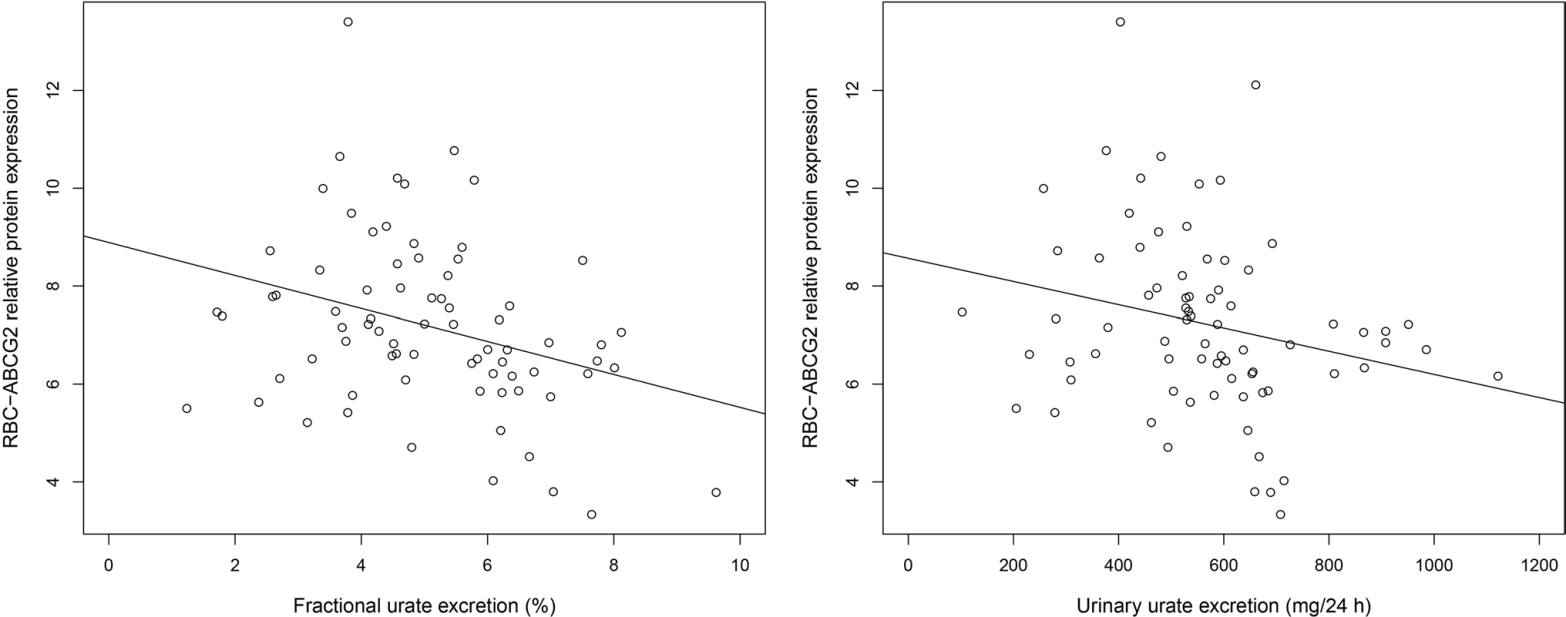
Correlation of renal-overload hyperuricemia clinical parameters and ABCG2 protein expression

The average FUE, UUE and erythrocyte ABCG2 protein expression level of gouty patients who had at least one ABCG2 SNP were significantly higher compared to gouty patients with no ABCG2 polymorphism (mean values ± SD and *p*-values see Table 2). Demonstrating the ABCG2 protein levels, Bxp34 levels showed significant negative correlation with FUE (Pearson correlation coefficient = −0.320, *p* = 0.005) and UUE (Pearson correlation coefficient = −0.241, *p* = 0.038). FUE and UUE showed strong positive correlation (Pearson correlation coefficient = 0.568, *p* < 0.001, Fig. 2a, b).

Correlation plot of fractional urate excretion (A) and urinary urate excretion (B) in regard to the relative protein expression of RBC-ABCG2.

## Discussion

Despite its importance in urate metabolism, only limited information is available on how clinical parameters correlate with ABCG2 protein expression and its genetic background. This study firstly aims to evaluate ABCG2 transporter levels with genetic polymorphisms and clinical data in gouty patients. Also, it focuses on how these changes may affect susceptibility and severity of the disease.

Our data shows that a substantial proportion (32.1%) of gouty patients carried the studied SNPs (Q141K, M71V, R236X or R383C) compared to the control population (13.7%), and that the presence of a functional mutation/variant significantly raised the possibility of presenting gout (OR 3.0) [33–35]. Early onset gout (age of 40 or under), especially with frequent flares and/or tophi formation are major characteristics of a severe disease that may lead to higher comorbidity rates [23, 24, 36]. Our results demonstrate that patients with ABCG2 functional polymorphisms presented gout approximately 8 years earlier than wild type gouty patients, on average at the age of 37.6. Moreover, we found that frequent flares (≥ 2/year) were also independently associated with the studied ABCG2 SNPs. The numbers of tophaceous cases were greater if these genetic changes were present, however it did not reach the significance level. These findings suggest that functional ABCG2 polymorphisms may prove to be a risk factor for an early onset, severe gout.

Prior to our method [25] that allows one to analyse ABCG2 protein expression from a single drop of blood, there was limited clinically relevant information on in vivo ABCG2 transporter protein expression and that data mostly relied on mouse models [37, 38]. Our results successfully proved that if the studied functional SNPs were present, ABCG2 transporter protein expressions showed a significant decrease. This underexpression was most critical if the M71V mutation was present, which emphasises the importance of this lately described loss of function variant.

As approximately 30% of urate excretion is conducted through the intestines [13], a decrease in extrarenal, mostly intestinal urate excretion may result in a renal-overload type hyperuricemia characterised by elevated values of FUE and UUE [37]. We found that both the altered protein levels and the genetic changes correlated with an inversely proportional shift of the studied FUE and UUE parameters, showing elevated (near normal) FUE and increased UUE levels. These results in vivo confirm previous data [37] that impaired ABCG2 transport function acted as an extra-renal cause of urate excretion deficiency leading to a renal-overload type hyperuricemia.

We are aware that this study also has limitations. Proper diet and hydration were controlled by self-reports and while the latter was checked during the general physical examination, it is hard to completely exclude all dietary bias. Strict sample management and high human resource needs resulted in relatively modest population numbers that should be further augmented. Also, according to the current EULAR guideline screening and treating comorbidity is a priority in gout management. Therefore disease severity should be evaluated along with comorbidity data. Urate synthesis inhibitors in general are favoured in ROL type hyperuricemia as well, however ABCG2 dysfunction might be linked with poor therapeutic response to the first drug of choice allopurinol [39–43]. The use of small molecule medication colchicine in vitro proved to be able to upregulate ABCG2 transporter expression, as we previously published [29]. Testing this colchicine effect in vivo could take us a step closer to a more personalised therapy of gouty arthritis.

## Conclusions

ABCG2 functional polymorphisms were associated with higher gout susceptibility and a clinically severe, early onset disease. Human in vivo RBC-ABCG2 protein expression levels were examined together with genetic and clinical backgrounds. The studied SNPs correlated with decreased protein expression which was furthermore associated with clinical data, such as elevated FUE and UUE values. These results show a clear link between ABCG2 dysfunction and ROL hyperuricemia in clinically defined gouty patients.

## Data Availability

The deidentified participant data generated and analysed in the current study is available on the open repository site zenodo.org.

## Abbreviations

GWAS: Genome-wide association study
ABCG2: ATP-binding cassette (ABC) transporter family (subgroup G2)
ROL (hyperuricemia): Renal overload hyperuricemia
FUE: Fractional urate excretion
UUE: Urinary urate excretion
RBC: Red blood cell
SU: Serum urate
qPCR: Quantitative polymerase chain reaction
SNP: Single nucleotide polymorphism
MAF: Minor allele frequency
